# Level of episiotomy practice and its disparity among primiparous and multiparous women in Ethiopia: a systematic review and meta-analysis

**DOI:** 10.3389/fgwh.2023.1153640

**Published:** 2023-11-06

**Authors:** Fantu Mamo Aragaw, Daniel Gashaneh Belay, Mastewal Endalew, Melaku Hunie Asratie, Moges Gashaw, Nuhamin Tesfa Tsega

**Affiliations:** ^1^ Department of Epidemiology and Biostatistics, Institute of Public Health, College of Medicine and Health Sciences, University of Gondar, Gondar, Ethiopia; ^2^ Department of Human Anatomy, College of Medicine and Health Sciences, University of Gondar, Gondar, Ethiopia; ^3^ Department of Environmental and Occupational Health and Safety, Institute of Public Health, College of Medicine and Health Sciences, University of Gondar, Gondar, Ethiopia; ^4^ Department of Women’s and Family Health, School of Midwifery, College of Medicine and Health Sciences, University of Gondar, Gondar, Ethiopia; ^5^ Department of Physiotherapy, College of Medicine and Health Sciences, University of Gondar, Gondar, Ethiopia

**Keywords:** episiotomy, practice, systematic review, meta-analysis, women

## Abstract

**Background:**

Episiotomy at the time of vaginal birth is a common lifesaving surgical procedure. In Ethiopia, several studies have been conducted concerning the proportion of episiotomy. However, its prevalence varies across these series of studies. Thus, this systematic review and meta-analysis aimed to estimate the level of episiotomy practice and its disparity among primiparous and multiparous women in Ethiopia.

**Methods:**

This systematic review was reported according to the Preferred Reporting Items for Systematic Reviews and Meta-Analysis (PRISMA) guideline. We systematically searched the PubMed/MEDLINE, EMBASE, Google Scholar, and Science Direct databases for studies conducted in Ethiopia focusing on episiotomy. We included all cross-sectional studies published until October 5,2022. Data were analyzed using R version 4.2.1 software. The pooled estimates with 95% confidence intervals (CIs) were presented using forest plots. A random-effects meta-analysis was conducted on extracted crude rates to calculate the national and regional pooled estimates for the country. The *I*-squared test and Egger's regression test were used to assess heterogeneity and publication bias, respectively.

**Results:**

Our search yielded 390 articles. A total of 13 studies covering five administrative regions and 6,404 women who delivered vaginally were involved. The mean age of the study participants ranged from 22 to 27.7 years. The estimated overall pooled prevalence rate of episiotomy in Ethiopian women was 42.75% (95% CI: 34.97%–50.54%). In the subgroup analysis, the pooled prevalence rate of episiotomy was 61.45% (95% CI: 51.11%–71.80%) among primiparous women. Meanwhile, the pooled estimate appears to be approximately 30.47% (95% CI: 22.08%–38.85%) among multiparous women.

**Conclusion:**

Our findings concluded that the pooled prevalence rate of episiotomy was higher than the evidence-based WHO recommendations for optimal patient care. Parallel to this, nulliparous women had a higher episiotomy rate than multiparous women. These findings highlight the importance of continued training for labor ward staff, particularly healthcare providers who often perform the majority of deliveries.

## Background

Episiotomy is one of the oldest surgical procedures involving the incision of the perineum to enlarge the vaginal opening during the second stage of labor (1, 2). Despite little scientific support for its routine use, it continues to be a frequently implemented obstetric procedure (3–5). Its prevalence varies across the globe, from almost a routine intervention in nearly all first births in some Latin American countries like Argentina (6), European countries (7), and the United States (8).

The major justification for the utilization of episiotomy is the prevention of severe perineal tears (9). Episiotomy prevents the occurrence of third-degree (involving the anal sphincter) and fourth-degree (involving the rectal mucosa) lacerations (9). An observational study has shown that episiotomy has a protective role during delivery (6). Despite the benefits of episiotomy, several studies have identified adverse consequences, including insufficient prevention of obstetric sphincter ani muscle injuries and hemorrhage (9–12). The practice of routine episiotomy increases the risk of major perineal injury (11).

Evidence reported that episiotomy rates vary according to parity (13). Previous studies have shown that primiparous women have an increased episiotomy rate compared with multiparous women (13–17). To date, the prevalence of episiotomy practice has been reported in numerous studies in Ethiopia, ranging from 25% to 65% (13–23). However, most of these studies did not determine the national-level prevalence. Moreover, these primary findings have been inconsistent and inconclusive. Hence, this systematic review and meta-analysis aimed to produce the pooled prevalence of episiotomy among women who delivered vaginally in Ethiopia. Furthermore, this comprehensive estimate will be important to support programmers, policymakers, and other stakeholders in making evidence-based decisions.

## Methods

### Evidence acquisition

An intensive search was performed in the PubMed/MEDLINE and EMBASE online databases to access articles on episiotomy practice in Ethiopia. Moreover, Google Scholar and Science Direct were used to retrieve articles. In addition, the reference lists of the screened studies were checked to ensure that all relevant studies were included in the systematic review. Three authors (FA, NT, and MG) independently performed the search. The term “episiotomy” was searched with all of the subsequent terms as a mix of free text and thesaurus terms in numerous variations: vaginal delivery, instrumental delivery, maternity care, and Ethiopia. This systematic review and meta-analysis followed the Preferred Reporting Items for Systematic Review and Meta-Analysis (PRISMA) guidelines (Supplementary Table S1).

### Inclusion and exclusion criteria

#### Inclusion criteria

The inclusion criteria included all studies conducted in Ethiopia, women who delivered vaginally, all published and unpublished articles, studies published in the English language, studies that employed an observational study design, and articles published until October 5, 2022.

#### Exclusion criteria

Articles for which we could not obtain the full text even after two email contacts with the principal investigator or the corresponding author of the particular study were excluded from the analysis.

### Study selection procedures

The EndNote X7 citation manager was used to import studies extracted from several sources and to remove duplicates. Three review authors (FA, ME, and NT) independently assessed the inclusion of all the potential studies identified as a result of the search strategy. Other review authors (DB, MA, and MG) assessed the full text of the articles for eligibility for the final inclusion in this study. Any disagreements were resolved through discussion. Finally, eligible articles with full texts were reviewed.

### Data extraction

We designed a form to extract data, and three review authors (FA, DB, and NT) extracted the data using the agreed Microsoft Excel form. Any discrepancies were resolved through a review involving the other three authors (ME, MA, and MG). Information on the study location, region, publication year, study design, sample size, name of the authors, and number of episiotomy cases among primiparous and multiparous women was extracted from each study (Supplementary Table S2).

### Study quality and validity

Two authors (FA and MA) independently assessed the quality of each original study using the Newcastle–Ottawa Scale for the quality assessment of cross-sectional studies (Supplementary Table S3). The quality of each study was assessed using the following criteria: representativeness of the study, adequate sample size, acceptable nonresponse rate, use of validated measurement tools, comparability of the study, description of the outcome assessment, and use of appropriate statistical tests. Articles with a global rating score ≥ 7 out of 10 were considered high quality. Any disagreements between the two reviewers were resolved through discussion.

### Outcome definition

Episiotomy is a surgical incision of the vaginal orifice and perineum to facilitate the passage of a fetus in a woman who gives birth vaginally (24).

### Evidence synthesis and analysis

Data were extracted using a standardized data form prepared in a Microsoft Excel spreadsheet and analyzed using R version 4.0.5 statistical software. The pooled estimates with 95% confidence intervals (CIs) were presented using forest plots. A random-effects meta-analysis was conducted on extracted crude rates to calculate the national and regional pooled estimates for the country. The *I*-squared test and Egger's test were used to assess heterogeneity and publication bias, respectively. For the random-effects model, the DerSimonian and Laird weights were used to estimate the pooled proportion.

### Heterogeneity across studies

Heterogeneity among reported proportions was assessed by computing *p*-values of the *I*-squared test (25). In this study, significant heterogeneity was observed among the included studies (*I*^2^ = 94.47%, *p* < 0.001). As a result, a random-effects meta-analysis model was used to estimate DerSimonian and Laird's pooled effects.

### Additional analysis

A subgroup analysis was performed to identify potential moderating factors that can explain the inconsistencies between effect sizes across the primary studies based on different variables (i.e., geographical settings and parity). In addition, a univariate meta-regression model was used by taking the sample size, publication year, and quality score of each study to investigate the sources of heterogeneity.

## Results

### Search results and study selection

**Figure 1:**
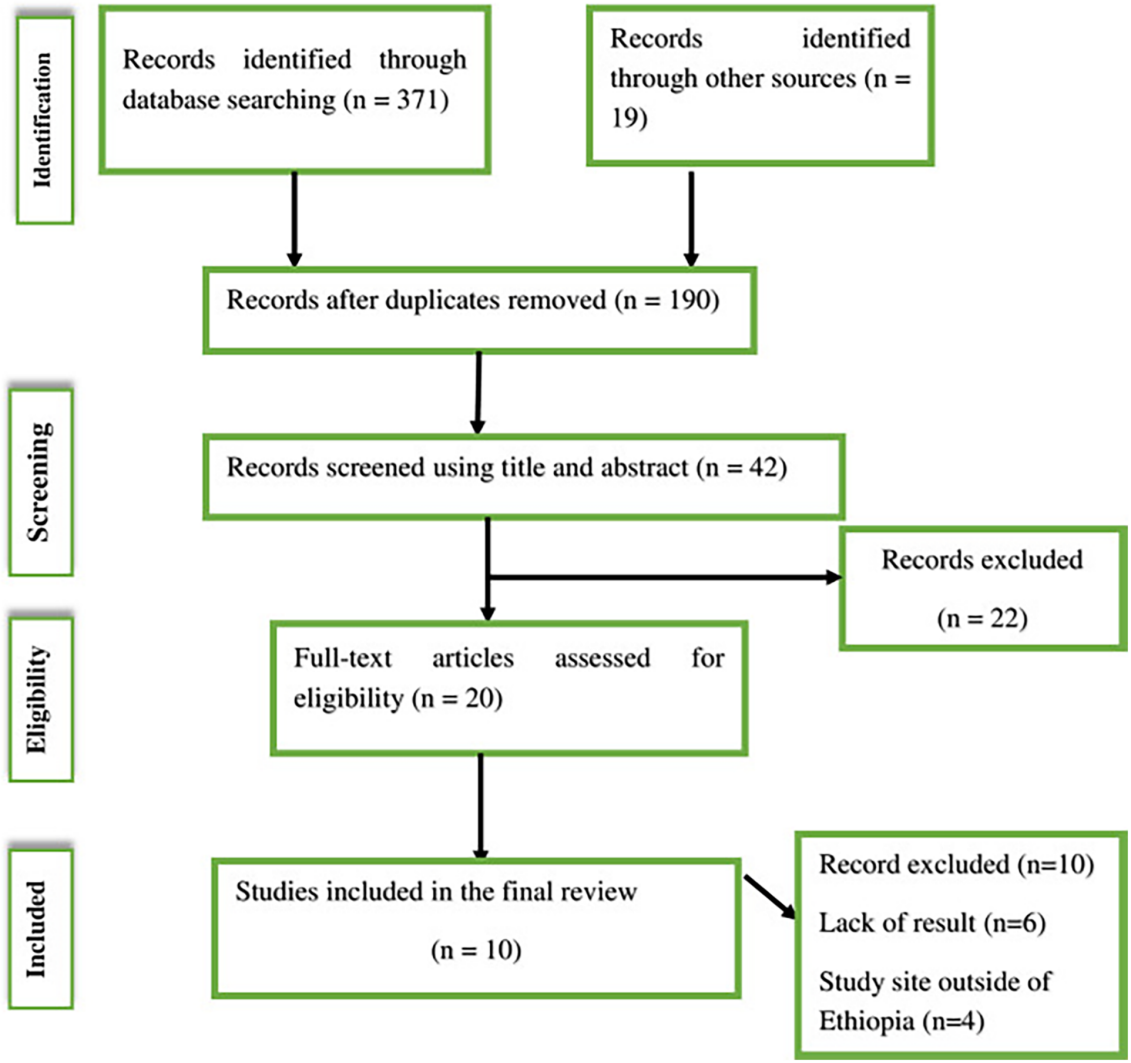
PRISMA flow diagram describing the selection of studies for a systematic review and meta-analysis of the prevalence of episiotomy among women who delivered vaginally in Ethiopia.

During our initial search, 390 articles were retrieved from PubMed, Google Scholar, the Cochrane Library, and Google for gray literature. Of these initial articles, 200 duplicate articles were excluded. Among the remaining 190 articles, 148 articles were excluded because of their titles and abstracts. After reviewing the abstracts, 22 articles were removed. Finally, 23 full-text articles were assessed for eligibility. Among them, 10 full-text articles were excluded due to the absence of results and study location. At the end of the process, 13 studies remained eligible and were included in the systematic review. Figure 1 describes the detailed selection procedures.

### Characteristics of original studies

Table 1 summarizes the study characteristics such as the year of publication, sample size, study design, and study area parameters of the nine included studies.

**Table 1:** Study characteristics of included articles for the systematic review and meta-analysis on the prevalence of episiotomy among women who delivered vaginally in Ethiopia.

No.	Authors	Year	Study area and facility type	Sample size	Prevalence of episiotomy (%)		
					Primiparous (%)	Multiparous(%)	Combined
1	Yemane et al. (14)	2017	Public Health Institutions of Axum Town (Tigray region)	338	48.7	31.5	41.4
2	Worku et al. (15)	2019	Public Health Institutions of Akaki Kality (Addis Ababa)	381	67.1	13.9	35.2
3	Teshome et al. (13)	2020	University of Gondar Comprehensive Specialized Referral Hospital (Amhara region)	306	54.9	29.9	47.7
4	Mitiku et al. (16)	2015	Mizan Aman General Hospital (southern region)	310	39.8	31.4	30.6
5	Niguse et al. (17)	2016	Public health facilities of Shire Town (Tigray region)	407	50	27.7	35.4
6	Kiroset al. (19)	2006	Tikur Anbessa Teaching Hospital (Addis Ababa)	672	–	–	40.2
7	Aynalem FW (26)	2016	Debre Markos Referral Hospital (Amhara region)	314	–	–	42
8	Marai (20)	2002	Jimma Teaching Hospital (Oromia region)	2,861	47	5	25
9	Woretaw et al. (27)	2020	Public Health facilities at Metema district (Amhara region)	410	–	–	44.15
10	Tefera et al. (21)	2019	Saint Paul's Hospital Millennium Medical College (Addis Ababa)	405	84.2	44.2	65.4
11	Beyene et al. (23)	2020	Felege Hiwot Referral Hospital (Amhara region)	411	64.9	27.2	41.1
12	Fikadu et al. (22)	2020	Arba Minch General Hospital (southern region)	410	80.66	53.72	68
13	Tamene et al. (28)	2020	St. Paul's Hospital (Addis Ababa)	344	61.86	16.00	41.9

In this systematic review, a total of 6,404 women who had vaginal delivery were included. The mean age of the study participants ranged from 22 to 27.7 years. Concerning the study design, all included studies were cross-sectional. The sample size of the individual studies included in our meta-analysis ranged from 306 (13) to 2,861 (20). Furthermore, all studies were conducted between 2002 and 2020. In this study, four Ethiopian regions and one administrative town were represented. Three of the studies were conducted in Addis Ababa city administration (15, 19, 21), four in the Amhara region (13, 18, 23, 29), two in the Tigray region (14, 17), two in the southern region (16, 22), and one in the Oromia region (20). No research has been found in Dire Dawa city administration and the Benishangul-Gumuz, Harari, Afar, Gambella, and Somali regions.

### Quality of the included studies

The quality score of the included studies ranged from 6 to 9 out of 10 total quality scores, with a mean quality score of 7.7 (SD ± 1.3) (Supplementary Table S3). Overall, studies with a quality score of ≥6 are considered high quality. Last, all 13 included articles were categorized as high-quality studies.

### Sensitivity analysis and publication bias

**Figure 2:**
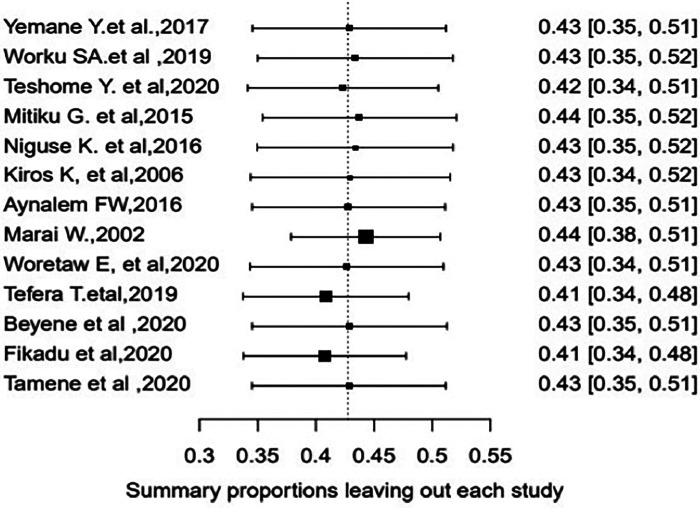
Leave-one-out sensitivity analysis for single-study influence on the pooled estimate of episiotomy among women who delivered vaginally in Ethiopia.

**Figure 3:**
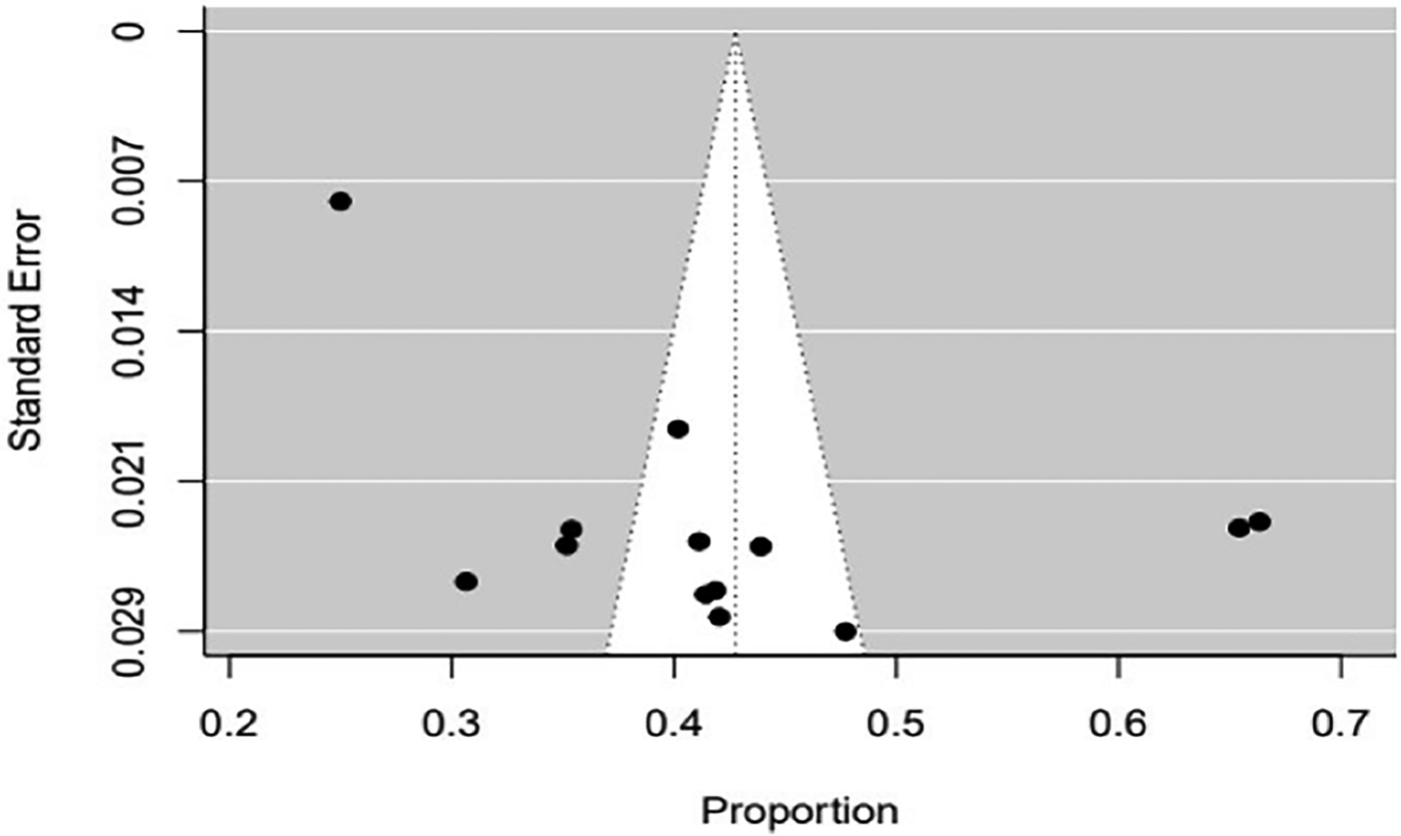
Funnel plot episiotomy practice among women who delivered vaginally in Ethiopia.

First, we screened articles for studentized residuals larger than two absolute values to identify outlying and influential studies. During the screening process, two articles (16, 21) seemed influential (Figure 2). Accordingly, a leave-one-out sensitivity analysis was performed to determine whether they were truly influential, which indicates that the exclusion of one article (21) had a significant change in the fitted meta-analytic model (Supplementary Figure S1). As a result of this considerable influence on the summary effect size, we eliminated this article (21) from the final meta-analysis. After the process, 13 studies were used in the final meta-analysis. Regarding the publication bias of the included articles, a funnel plot and Egger's regression test were used to check the indication of publication bias. As indicated in Figure 3, there is clear evidence of heterogeneity and funnel plot asymmetry. Despite this clear evidence in the funnel plot, Egger's regression test failed to find a significant publication bias (*p* = 0.56).

### Meta-analysis

**Figure 4:**
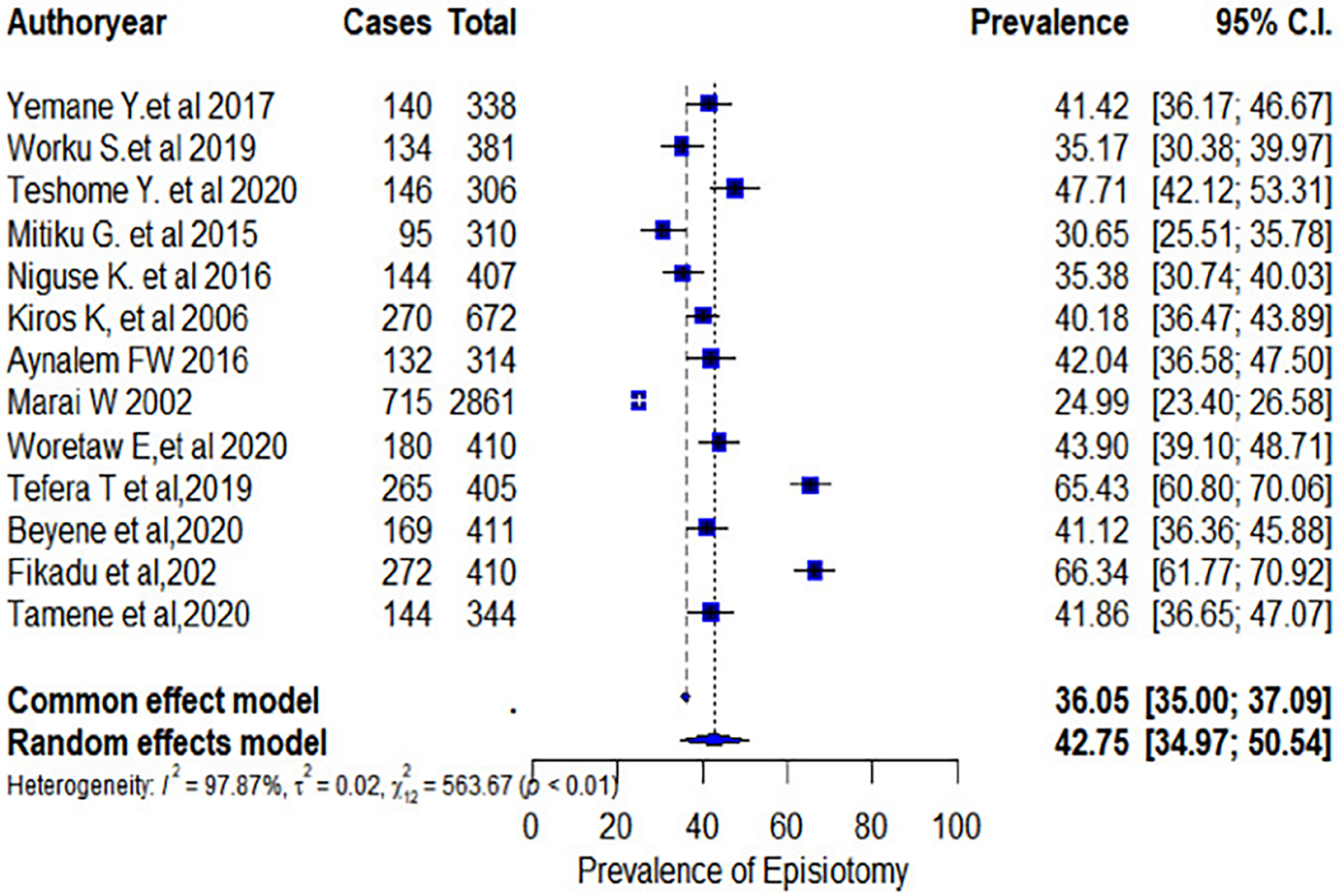
Forest plot of the pooled prevalence of episiotomy among women who delivered vaginally in Ethiopia.

The overall estimated pooled prevalence rate of episiotomy among women who delivered vaginally in Ethiopia was 42.75% (95% CI: 34.97%–50.54%) (Figure 4). In addition, the forest plot of our meta-analysis showed that the highest proportion (47.71%) of episiotomy was reported from a study conducted at the University of Gondar Comprehensive Specialized Referral Hospital in Amhara Regional State (13). In contrast, the lowest proportion (25%) was reported from a study at Jimma University Specialized Hospital in Oromo Regional State (20). According to the *I*-squared test statistics (*I*^2 ^= 95.47%), the included studies showed high heterogeneity. Therefore, subgroup and univariate meta-regression analyses were conducted to identify the possible sources of heterogeneity.

### Subgroup analysis

**Figure 5:**
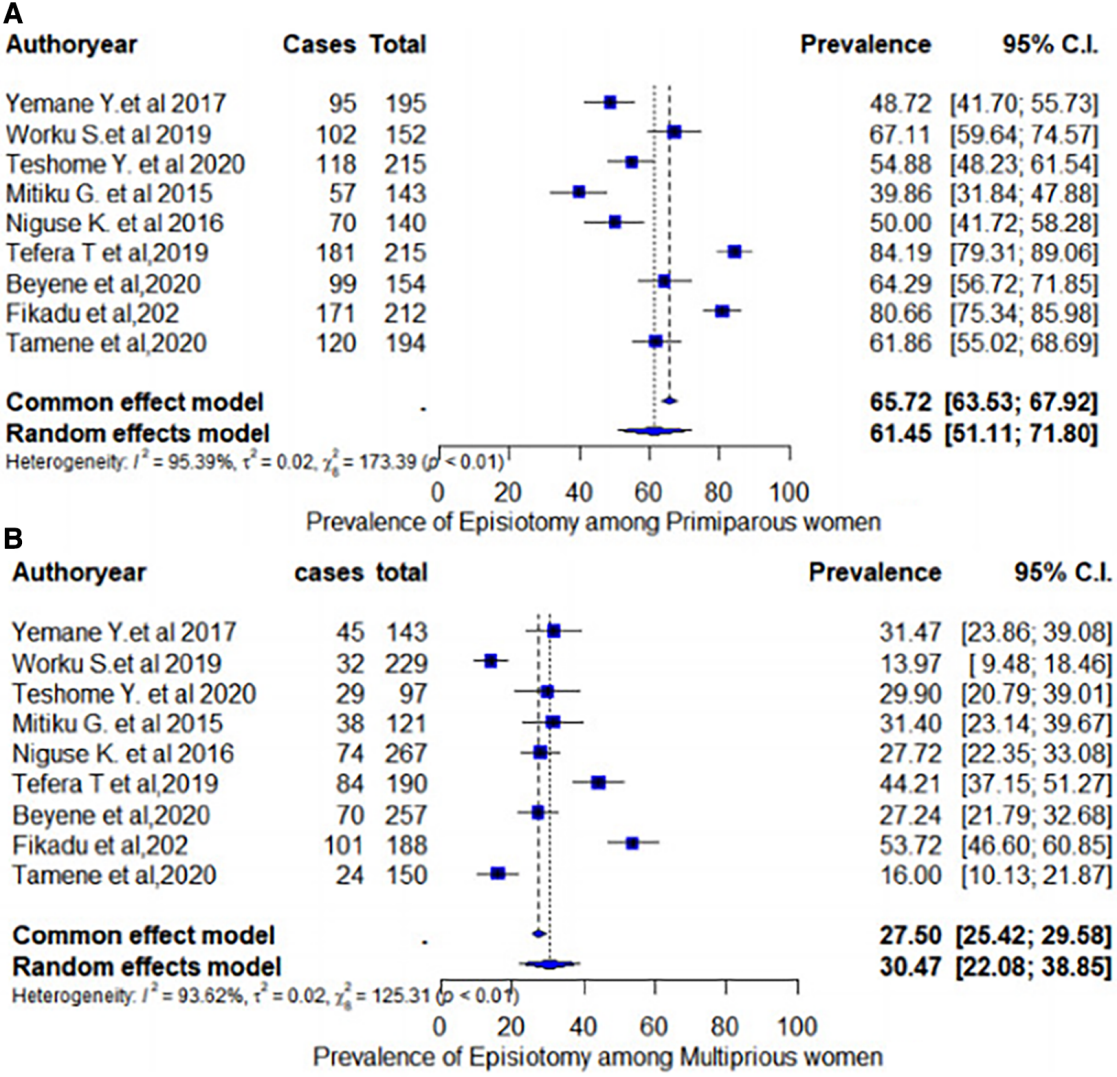
Pooled prevalence of episiotomy among women who delivered vaginally in Ethiopia by parity: (**A**) pooled prevalence of episiotomy among primiparous women; (**B**) pooled prevalence of episiotomy among multiparous women.

**Figure 6:**
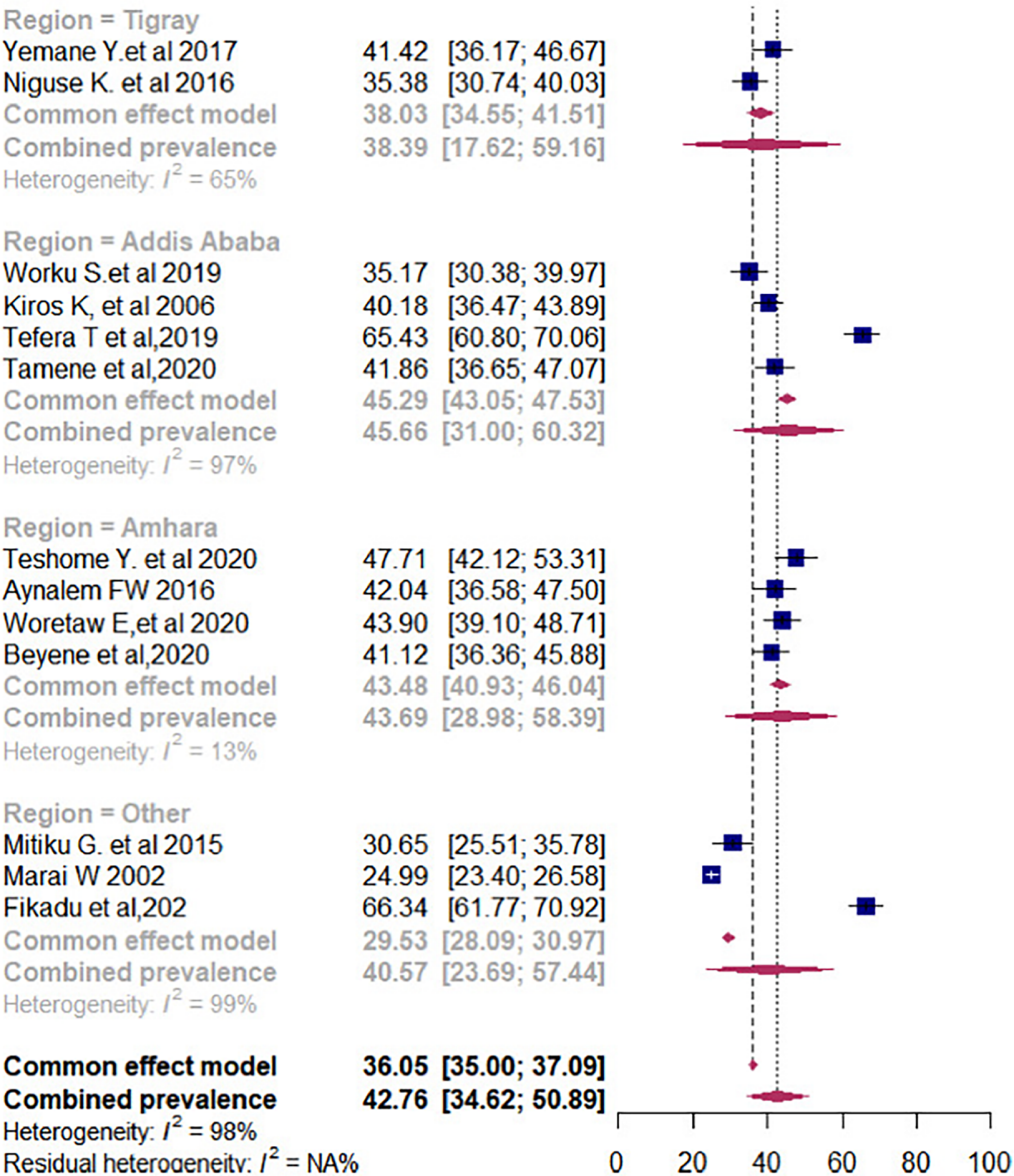
Pooled prevalence of episiotomy among women who delivered vaginally in Ethiopia by regions.

The subgroup prevalence of episiotomy was estimated by considering the parity and geographical settings of women who delivered vaginally. In the case of heterogeneity, we have conducted a subgroup analysis by considering parity (primiparous or multiparous) to explore possible causes. Accordingly, in the subgroup analysis, the pooled prevalence rate of episiotomy among primiparous women was 61.45% (95% CI: 51.11%–71.80%) (Figure 5A). In contrast, the pooled estimate of episiotomy among multiparous women was 30.47% (95% CI: 22.08%–38.85%) (Figure 5B). In addition, the pooled prevalence of episiotomy varied widely across the regions of Ethiopia. As such, in the regional subgroup analysis, the Amhara region had the highest pooled proportion of episiotomy practice at 44.59% (95% CI: 44.15%–48.85%), followed by the Tigray region at 38.23% (95% CI: 33.07%–40.40%), and Addis Ababa city administration at 37.90% (95% CI: 33.07%–42.76%) (Figure 6).

### Meta-regression

A meta-regression was conducted using the study as the unit of analysis to examine the relationship between covariates (sample size, publication year, and quality score of primary studies) and the effect sizes (Table 2). From this output, we can conclude that none of the covariates significantly affected the pooled estimate.

**Table 2:** Meta-regressions of the episiotomy practice among women who delivered vaginally in Ethiopia by sample size, publication year, and quality scores of included studies.

Covariate	β (95% CI)	*p*-value
Publication year	0.0022 (−0.0096 to 0.0139)	0.7180
Sample size	0.0001 (−0.0001 to 0.0002)	0.3843
Quality score	−0.0045 (−0.0507 to 0.0416)	0.8468

## Discussion

This systematic review and meta-analysis aimed to estimate the pooled prevalence of episiotomy among Ethiopian women who delivered vaginally. As per the results of this meta-analysis, the pooled prevalence rate of episiotomy among women who delivered vaginally was 42.75% (95% CI: 34.97%–50.54%). This rate is in line with studies conducted at the Nigeria tertiary hospitals at 40.4% (30), Brigham Hospital Boston Massachusetts at 40.6% (31), Morocco at 41.28% (32), and Iran at 41.5% (33). On this basis, even though the exact rate of episiotomy is unknown, it is appropriate to conclude that the episiotomy rate is still higher. Episiotomy was performed to help prevent severe vaginal tears during delivery. However, more recent research suggests that an episiotomy may cause more problems than it aims to prevent. The procedure can increase the risk of infection and other complications. Recovery also tends to be lengthy and uncomfortable (2, 34, 35).

However, episiotomy rates vary worldwide. Our pooled estimate of episiotomy prevalence was higher than that of the World Health Organization recommendation (5%–15%) (36). Furthermore, our result (37.82%) was significantly higher than that of other countries such as Denmark (3.7%) (37), Congo (20.4%) (38), and Brazil (29.1%) (39). This overuse of episiotomy intervention might be a symptom of the obstetric transition with medicalization and increasing interventionist birth practices with the obstetric transition stage. Obstetric transition refers to the long-term trend of countries gradually shifting from a pattern of high maternal mortality to a pattern of low maternal mortality; from a direct obstetric cause of maternal mortality to an increasing percentage of indirect causes, non-communicable causes, and maternal population aging; and from the natural course of pregnancy and childbirth to the institutionalization of maternity care, increasing the rates of obstetrical interruption (40). It is the process of transforming a population's reproductive health and birth patterns through time (41). In addition, women in low-income settings are often not informed about the risks and reasons for interventions and not asked to provide informed consent (42). This might contribute to the increments in the episiotomy rate in Ethiopia. Moreover, this wide practice variation suggests that episiotomy use is heavily driven by local professional norms, experiences in training, and individual practitioner preference rather than variations in the needs of individual women at the time of vaginal birth.

Performing episiotomies without consent during labor and childbirth has been deemed disrespectful and abuse or obstetric violence (43). Therefore, it is necessary to obtain informed consent before performing episiotomy during childbirth (44, 45). Selective episiotomies are significantly less likely to be associated with accusations of obstetric violence during childbirth than routine episiotomies (44, 46). Healthcare providers should avoid performing unnecessary episiotomies and understand the potential risks associated with this procedure (45, 46).

Our chief findings suggested that nulliparous women had a higher episiotomy rate (52%) than multiparous women (26.51%). Earlier evidence supported this finding (5, 7, 47). This may be explained by the plausible reason that primiparous women often have a tight perineum, which is one indication of episiotomy, and the old recommendation of routine episiotomy in primiparous women performed by many health professionals might still have an influence on the indication of this procedure for those women. Furthermore, this finding is probably because many healthcare providers still practice the policy of preventing uncontrolled perineal tears by performing episiotomies on nulliparous women. This study also demonstrates that the pooled prevalence of episiotomy varied across regions of Ethiopia. Accordingly, the Amhara region had the highest pooled proportion of episiotomy practice at 44.59% (95% CI: 44.15%–48.85%). This regional variation might be due to the variations in medical practices that exist among the regions.

## Limitations

Tremendous efforts have been made to include all articles from Ethiopia. However, this study was not free from limitations. Only articles published in English were considered. Moreover, we have not obtained studies from Benishangul-Gumuz, Ethio-Somali, Afar, Dire Dawa city administration, and Gambella region, which might affect the issue of generalizability.

## Conclusions

Our findings concluded that the pooled prevalence of episiotomy was higher than the evidence-based WHO recommendations for optimal patient care. Parallel to this, nulliparous women had a higher episiotomy rate than multiparous women. These findings highlight the importance of continued training for labor ward staff, particularly healthcare providers, who often perform most deliveries.

## Data Availability

The original contributions presented in the study are included in the article/**Supplementary Material**, further inquiries can be directed to the corresponding author.

## Supplementary material

Supplementary Table S1 PRISMA 2009 checklist. (DOC).

Supplementary Table S2 Data extraction sheet (XLSX).

Supplementary Table S3 Quality score of each study (XLSX) zcx.

Supplementary Figure S1 Plots of diagnosis (PDF).
