# Supplementary figures and images for: Level of episiotomy practice and its disparity among primiparous and multiparous women in Ethiopia: a systematic review and meta-analysis
Source: Front Glob Womens Health. 2023 Nov 6;4:1153640. doi: 10.3389/fgwh.2023.1153640 (PMC10657876; doi:10.3389/fgwh.2023.1153640)

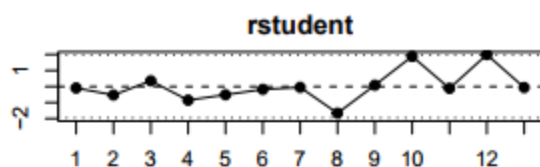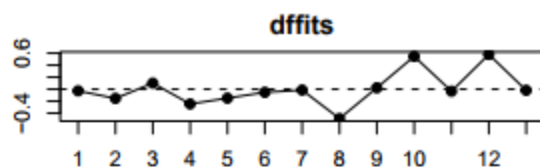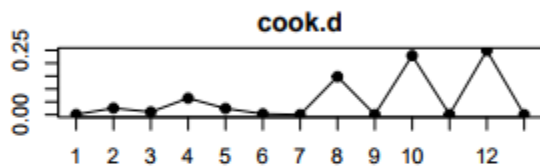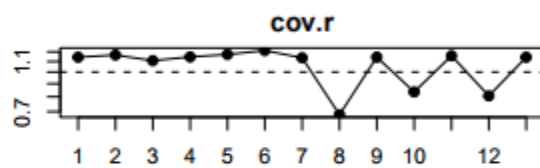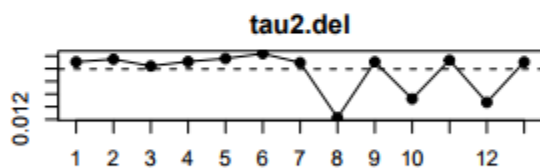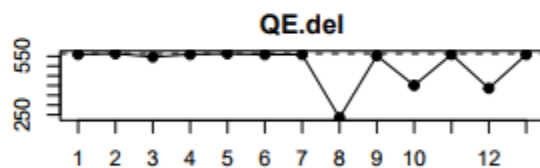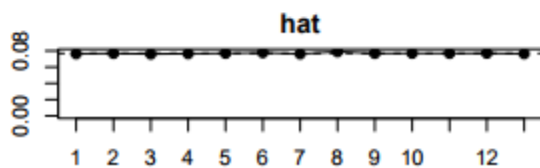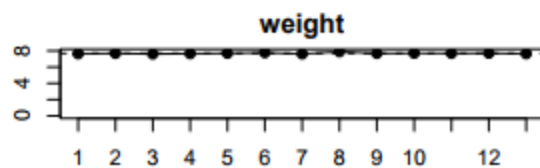

Supplement: Supplementary Figure S1 [file Image1.pdf]
